# Early and Midterm Major Adverse Cardiac Events in Patient With Saphenous Vein Graft Using Direct Stenting or Embolic Protection Device Stenting

**DOI:** 10.5812/cardiovascmed.13012

**Published:** 2014-02-24

**Authors:** Mohammadali Sadr-Ameli, Hossein Mousavi, Mona Heidarali, Mohsen Maadani, Yones Ghelich, Behshid Ghadrdoost

**Affiliations:** 1 Cardiac Electrophysiology Research Center, Rajaie Cardiovascular Medical and Research Center, Iran University of Medical Sciences, Tehran, IR Iran; 2 Rajaie Cardiovascular Medical and Research Center, Iran University of Medical Sciences, Tehran, IR Iran; 3 Cardiovascular Intervention Research Center, Rajaie Cardiovascular Medical and Research Center, Iran University of Medical Sciences, Tehran, IR Iran; 4 Department of Intervention, 502 Military Hospital, Tehran, IR Iran

**Keywords:** Lesion, Blood Vessels, Saphenous Vein, Transplants, Embolic Protection Devices, Coronary Artery Bypass

## Abstract

**Background:**

The treatment of an occluded saphenous vein graft (SVG) with percutaneous coronary intervention may encounter major adverse cardiac events (MACE). MACE rates have been reduced significantly by using the embolic protection device (EPD).

**Objectives:**

The aim of this study was to clarify the risks and the benefits of embolic protection devices.

**Patients and Methods:**

In a prospective, non-randomized observational study, patients aged 33 to 85 years old who underwent elective percutaneous coronary intervention due to SVG stenosis at our tertiary care center were enrolled between 2009 and 2011. The incidence rates of adverse events, including MACE, were obtained during the patients’ hospitalization and at 30-day and 6-month follow-up. MACE included death, Q-wave and non-Q-wave myocardial infarction, in-stent thrombosis, target lesion revascularization, and target vessel revascularization.

**Results:**

From 150 patients enrolled to the study, 128 (85.3%) patients underwent direct stenting and the rest underwent the EPD procedure. In-hospital MACE occurred in 17.2% of the patients in the direct stenting group versus only 9.1% in the EPD group (P = 0.530). MACE incidence was gradually increased at one and 6-month follow-up periods in the direct stenting group (19.5% and 21.9%, respectively), and remained unchanged in the EPD group (9.1% at six-month follow-up). Multivariate logistic regression model showed that the stenting procedure type could not predict early and midterm MACE with the presence of baseline characteristics as cofounders.

**Conclusions:**

Despite the considerable lower early and midterm MACE rates, numerically following the EPD procedure compared to direct stenting, the difference in the MACE rates between the two groups was not significant.

## 1. Introduction

Saphenous vein graft (SVG) percutaneous coronary intervention (PCI) carries unique technical challenges, requires the use of the embolic protection device (EPD) to reduce the adverse events associated with distal embolization. Distal embolization is a common and almost omnipresent consequence of SVG PCI due to the soft and friable nature of SVG lesions (1). Adequate myocardial reperfusion, and therefore acceptable functional recovery may not be achieved with traditional PCI which could be due to ischemia or the distal embolization of plaque or thrombus material from the target lesion (2). Using distal EPD can reduce the complication rate of PCI by allowing the collection and removal of embolic debris (3). Several types of these devices have been developed to improve clinical outcomes by removing thrombi and to protect against distal embolization during PCI (4). Some studies have shown the beneficial effects of using these protection devices (5-8). More recently, larger randomized controlled trials have evaluated major adverse cardiac events (MACE) as an endpoint, and followed patients after hospital discharge; these studies have, nevertheless, yielded conflicting results (2, 9-12). Consequently, comparative efficacy and safety of these devices in comparison with traditional methods not only are unclear but also require further evaluation.

## 2. Objectives

There are reports on the safety and efficacy of direct stenting in SVG lesions in the current study. Therefore, the present study draws a direct comparison between the two techniques. Our objective was to perform a comparative effectiveness study to examine the benefits associated with using EPD to remove thrombi or protect against distal embolization in patients underwent occluded SVG PCI of occluded saphenous vein graft (SVG).

## 3. Patients and Methods

Data was collected prospectively on a cohort of patients underwent PCI on the SVG at our tertiary care center from 2009 to 2011. The inclusion criteria was SVG occlusion confirmed by coronary angiography, and the exclusion criteria included refusal to continue the study, occurrence of noncardiac adverse events leading to death within the study protocol, and presentation of acute coronary syndrome. Baseline measurements included demographics, cardiac history, graft age, and stent type. The patients were treated with one of the two methods of direct stenting or EPD. The study was conducted in accordance with the declaration of Helsinki protocol and was approved by the Ethics committee of Tehran University of Medical Sciences. All the patients provided written and informed consent prior to entering the study. Information on adverse events - including MACE - was obtained during the patients’ hospitalization and at 30-day and six-month follow-up periods. MACE included death, Q-wave and non-Q-wave myocardial infarction, in-stent thrombosis, target lesion revascularization, and target vessel revascularization. The follow-up visits were performed either by the referring cardiologist or alternatively via telephone contact.

The results were reported as mean ± standard deviation (SD) for the quantitative variables and percentages for the categorical variables. The groups were compared using Student's t-test for the continuous variables and chi-squared test (or Fisher's exact test if required) for the categorical variables. Predictors exhibiting a statistically significant relation with MACE in the two groups in univariate analyses were taken for multivariate logistic regression analysis to investigate their independence as predictors. Odds ratios (ORs) and 95% confidence intervals (CIs) were calculated. P values ≤ 0.05 were considered statistically significant. All the statistical analyses were performed using SPSS (version 13.0 for Windows, SPSS Inc., Chicago, IL, the USA).

## 4. Results

The study population consisted of 150 patients with a mean age of 63.23 ± 9.53 years. Direct stenting was performed in 128 (85.3%) patients, and 22 (14.7%) of the patients underwent the EPD procedure. Stent type in the direct stenting and EPD groups were respectively 18.8% and 9.1% drug-eluting stents versus 81.3% and 90% bare-metal stents; there was no statistically significant association between the two groups (P = 0.3). The in-hospital mortality rate was two patients at one-month follow-up, and two patients at six-month follow-up in the direct stenting group compared to none in the EPD group at the same time points. In-hospital thrombosis occurred in one case at one-month follow-up and in 3 cases at 6-month follow-up in the direct stenting group compared to none in the EPD group at the same points in time. In the direct stenting group, there was no case of target vessel or target lesion revascularization at one-month follow-up, but there was one case of target vessel revascularization and five cases of target lesion revascularization at six-month follow-up. In the EDP group, there were no cases of target vessel or target lesion revascularization at one and 6-month follow-up periods. No significant difference was found between the two groups regarding gender distribution and mean age (P > 0.05). Except for current smoking which was more prevalent in the EPD group (54.5%) (P = 0.001), the prevalence of coronary disease risk factors including history of hypertension, hyperlipidemia, diabetes mellitus, and family history of coronary artery disease were similar between the two study groups (Table 1). The mean SVG age was also comparable between the direct stenting and EPD groups (9.23 ± 4.80 vs. 8.59 ± 3.74; P = 0.556). In the direct stenting group, 18.8% of the patients received drug-eluting stents and 81.3% received bare metal stents, while the type of stents applied in the EPD group was drug-eluting in 9.1% and bare metal in 90.9% of the patients. No difference was found for stent type between the groups (P = 0.369). As regards in-hospital cardiac events (Table 2), in-hospital MACE occurred in 22 (17.2%) patients in the direct stenting group and only two (9.1%) patients in the EPD group (P = 0.53).

**Table 1.** Baseline Information of Study Subjects

Items	DS ^a^ Group, No. (%), n = 128	EPD^a^Group, No. (%), n = 22	P value
**Male**	93 (72.7)	19 (86.4)	0.17
**Age, Mean ± SD**	62.53 ± 8.65	63.23 ± 9.3	0.75
**Hypertension**	81 (63.3)	12 (54.5)	0.43
**Hyperlipidemia**	64 (50.0)	14 (63.6)	0.23
**Diabetes mellitus**	55 (43.0)	7 (31.8)	0.32
**Family history**	12 (9.4)	3 (13.6)	0.53
**Current smoking**	26 (20.3)	12 (54.5)	0.001
**Age of graft, Mean ± SD**	9.23 ± 4.08	8.59 ± 3.74	0.55
**DES** ^**a**^	24 (18.8)	2 (9.1)	0.17
**BMS** ^**a**^	104 (81.3)	20 (90.9)	0.17

^a^ Abbreviations: BMS, bare metal stent; DES, drug eluting stent; DM, diabetic mellitus; EPD, embolic protection device.

**Table 2.** Complications and Major Adverse Cardiac Events Following Study Procedures

Items	DS ^a^ Group, No. (%), n = 128	EPD ^a^ Group, No. (%), n = 22	P value
**In-hospital**			
Death	1 (0.8)	0 (0.0)	0.99
Myocardial infarction	20 (15.6)	2 (9.1)	0.53
Stent thrombosis	1 (0.8)	0 (0.0)	0.99
MACE ^a^	22 (17.2)	2 (9.1)	0.53
**One-month follow-up**			
Death	2 (1.6)	0 (0.0)	0.99
Myocardial infarction	23 (18.0)	2 (9.1)	0.53
Stent thrombosis	3 (2.3)	0 (0.0)	0.99
MACE	25 (19.5)	2 (9.1)	0.36
**Six-month follow-up**			
Death	2 (1.6)	0 (0.0)	0.99
Myocardial infarction	25 (19.5)	2 (9.1)	0.53
Stent thrombosis	3 (2.3)	0 (0.0)	0.99
TLR ^a^	5 (3.9)	0 (0.0)	0.999
TVR^a^	1 (0.8)	0 (0.0)	0.99
MACE	28 (21.9)	2 (9.1)	0.24

^a^ Abbreviations: DS, drug elution stent; EPD, embolic protection device; MACE, major adverse cardiac events; TLR, target lesion revascularization; TVR, target vessels revascularization.

**Figure 1.**
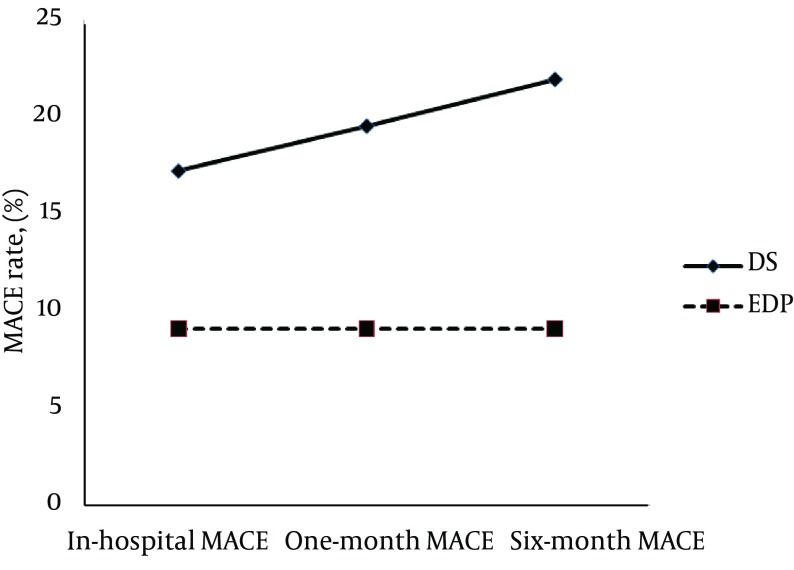
Trend of the Major Adverse Cardiac Events Over a Follow-Up Period of Six Months in Two Study Groups

Assessment of the MACE rates showed a gradual increase in the direct stenting group (19.5% and 21.9%, respectively) and no change in the EPD group at one-month and six-month follow-up (Figure 1). In the direct stenting group, no significant association was found between in-hospital and one-month MACE and baseline information (e.g. demographic data and medical history). No association was found between 6-month MACE and baseline data, except for a history of diabetes, which was associated with a higher midterm MACE rate in comparison with non-diabetics: 17 (60.7%) in the diabetic group and 38.0% in the non-diabetic group (P = 0.03). In the EPD group, none of the basic indicators was related to early and midterm MACE. In both groups, graft age and stent type were not associated with early and midterm MACE. Multivariate logistic regression model was used to exclude confounding factors (Table 3), and revealed that the type of stenting procedure could not predict midterm MACE with the presence of baseline characteristics as cofounders. There was no correlation regarding the MACE variables between the two groups (Table 3).

**Table 3.** Results of Logistic Regression Model for the Two Study Procedures and other Variables

Items	P value	Odds ratio	Confidence Interval, 95%
**Direct stenting**	0.346	2.152	0.437 - 10.596
**Male**	0.060	0.358	0.122 - 1.045
**Age, y**	0.192	1.036	0.982 - 1.093
**Hypertension**	0.181	1.843	0.892 - 4.515
**Hyperlipidemia**	0.796	0.886	0.354 - 2.217
**Diabetes mellitus**	0.450	1.469	0.542 - 3.981
**Family history**	0.307	1.819	0.578 - 5.728
**Current smoking**	0.170	0.225	0.027 - 1.889
**Age of graft **	0.768	0.986	0.895 - 1.085
**DES ** ^**a**^	0.266	1.815	0.635 - 5.190

^a^ Abbreviation: DES, drug eluting stent.

## 5. Discussion

Performing PCI on the SVG is associated with a high risk of MACE, mainly periprocedural myocardial infarction resulting predominantly from distal embolization of atherosclerotic plaque and friable debris within the graft and causing microvascular occlusion and no reflow (13-17). The EPD has been used as an adjunct to SVG PCI to reduce the occurrence of periprocedural events by approximately 40% (10). Although this represents a significant relative and absolute reduction in adverse events for one of the highest-risk subsets of PCI, the rate of adverse periprocedural events remains high even by using embolic protection, and these periprocedural events are allied to significant morbidity and economic cost (18, 19). Consequently, recognition of patients at the highest risk for periprocedural complications can confer appropriate risk stratification of patients before SVG PCI (21). These acute procedural complications render the long-term clinical outcome poor (18, 20, 21).

Since 2002, 4000 patients have been enrolled in studies evaluating the EPD (19, 22, 23). These studies have demonstrated that the rates of adverse events in the active treatment, range from 3.8% to 11.6% (24). These studies have also implicated older graft age (25-27) and angiographic characteristics such as presence of thrombus (20), lesion length (28), and diffuseness of the disease (28) as the predictors of adverse events. The most recent studies of late (1 - 3 years) outcomes for patients underwent SVG PCI are retrospective, span a period of time when therapies were evolving (1990 - 1998), and are contradictory with respect to conclusions relating event-free survival and stenting (25, 26). In the present study, the graft age was higher in the direct stenting group than the EPD group (9.23 ± 0.08 vs. 8.59 ± 3.74), but the difference between the two groups did not constitute statistical significance (P = 0.5). Elsewhere in a study, the graft age was 12 years and no association was found between adverse outcome and graft age (10). The age range was nearly similar in our direct stenting and the EDP groups (62.58 ± 8.65 vs. 63.23 ± 9.3, respectively), but the difference was not significant (P = 0.75). There was a higher rate of sex-influenced dominance in the EPD group by comparing with the direct stenting group, with males accounting for 86.4% of the 22 patients in the former group. However, there was no significant difference between the two groups regarding sex (P = 0.7).

In-hospital stent thrombosis occurred in 20 patients (15.6%) of the direct stenting group (n = 128), and none of the EPD group patients (P = 0.9). In one study, the mean patient age was 69 years, including 82% male and 41% diabetic subjects; these findings are consistent with our results (10). We evaluated early and midterm outcomes of patients underwent one of the two PCI procedures of direct stenting or EPD. In-hospital myocardial infarction was more frequent in the direct stenting group, 20 (15.6%) than the EPD group; but the difference between the two groups was not statistically significant (P = 0.53). One-month follow-up showed a higher incidence rate of myocardial infarction in the direct stenting group, 23 (18.5%) than the EPD group, which the difference between the two groups was not statistically significant (P = 0.53). Three (2.3%) cases of stent thrombosis were reported in the direct stenting group, but the difference between the two groups was not statistically significant (P = 0.9). Six-month follow-up of the patients in the direct stenting group revealed that myocardial infarction, 25 (19.5%) and stent thrombosis (2.3%) occurred more frequently in the direct stenting group; however, the difference was not statistically significant (P = 0.32) (Table 2). 

Diabetes mellitus is generally associated with a higher risk of adverse events after PCI. In one study, the diabetic patients were significantly younger, had lower SVG degeneration scores, and had smaller estimated plaque volumes by compared to non-diabetic subjects; these differences may account for the lower event rates among the former group in that data set (27). In our study, diabetes mellitus was reported by 55 (43%) of the 128 patients in the direct stenting group as opposed to 7 (31.8%) of the 22 patients in the EPD group; nonetheless, no statistically significant difference was found between the two groups (P = 0.3). A history of cigarette smoking is deemed a baseline covariate associated with MACE. Our results showed more current smoking in the EDP group (54.5%) in comparison to the direct stenting group (20.3%) (P = 0.001).

Several risk factors, including thrombus and graft age, have been described for SVG PCI without distal embolic protection (10). Interestingly, we did not observe any association between adverse outcomes and graft age, thrombus, or any other angiographic graft characteristics and none of the baseline indicators could predict midterm MACE. In fact, other variables such as intraoperative indicators and different technical aspects might be the predictors of midterm MACE, that should be assessed in future studies. In-hospital MACE occurred in 17.2% of the direct stenting group and in only 9.1% of the EPD group (P = 0.53). The most prevalent type of stent used in the both direct stenting and EPD groups was the bare metal stent (81.3% and 90%, respectively), but statistical difference was found between the two study groups (P = 0.3).

We finally evaluated the predictive value of baseline indicators in predicting midterm MACE related to PCI on the SVG, and our results confirmed that only 14.7% of the entire study population received EPD stenting, while most of them received direct stents. Although the MACE rate at different time periods was higher in the direct stenting group compared to the EPD group, but this difference was not statistically significant. Although the MACE rate was gradually increased during follow-up in the direct stenting group, it remained unchanged in the EPD group. Since patients’ survival can be manifested by the MACE indicator, it can be concluded that the EPD procedure can be more appropriate than direct stenting to improve patients’ survival and is, thus superior to the latter procedure. Some investigators have emphasized the superiority of direct stenting over the EPD procedure due to its more cost-effectiveness and availability, while some others have reported similar or higher effectiveness in the EPD procedure. These discrepancies can be due to the type of study (observational or trial), sample size or inclusion criteria for patient selection. Accordingly, clinical trials with greater sample sizes are required to reach reliable conclusions and compare direct stenting and EPD regarding reducing MACE following SVG PCI.

Identifying the predictors of MACE allows reliable prediction of patient outcomes and confirms consistent treatment benefits by using the EPD across the range of patients tested in randomized trials. Despite considerable lower early and midterm MACE rates following the EPD procedure compared to direct stenting, the difference in the MACE rates between our two study groups was not statistically significant. More studies with greater sample sizes are needed to confirm our results. The number of patients included in the EPD group was relatively low, partly due to the scarcity of such devices. Nevertheless, the results of the present study require further consideration.
